# Mitochondrial division/mitophagy inhibitor (Mdivi) Ameliorates Pressure Overload Induced Heart Failure

**DOI:** 10.1371/journal.pone.0032388

**Published:** 2012-03-27

**Authors:** Srikanth Givvimani, Charu Munjal, Neetu Tyagi, Utpal Sen, Naira Metreveli, Suresh C. Tyagi

**Affiliations:** Department of Physiology and Biophysics, University of Louisville School of Medicine, Louisville, Kentucky, United States of America; University of Frankfurt - University Hospital Frankfurt, Germany

## Abstract

**Background:**

We have previously reported the role of anti-angiogenic factors in inducing the transition from compensatory cardiac hypertrophy to heart failure and the significance of MMP-9 and TIMP-3 in promoting this process during pressure overload hemodynamic stress. Several studies reported the evidence of cardiac autophagy, involving removal of cellular organelles like mitochondria (mitophagy), peroxisomes etc., in the pathogenesis of heart failure. However, little is known regarding the therapeutic role of mitochondrial division inhibitor (Mdivi) in the pressure overload induced heart failure. We hypothesize that treatment with mitochondrial division inhibitor (Mdivi) inhibits abnormal mitophagy in a pressure overload heart and thus ameliorates heart failure condition.

**Materials and Methods:**

To verify this, ascending aortic banding was done in wild type mice to create pressure overload induced heart failure and then treated with Mdivi and compared with vehicle treated controls.

**Results:**

Expression of MMP-2, vascular endothelial growth factor, CD31, was increased, while expression of anti angiogenic factors like endostatin and angiostatin along with MMP-9, TIMP-3 was reduced in Mdivi treated AB 8 weeks mice compared to vehicle treated controls. Expression of mitophagy markers like LC3 and p62 was decreased in Mdivi treated mice compared to controls. Cardiac functional status assessed by echocardiography showed improvement and there is also a decrease in the deposition of fibrosis in Mdivi treated mice compared to controls.

**Conclusion:**

Above results suggest that Mdivi inhibits the abnormal cardiac mitophagy response during sustained pressure overload stress and propose the novel therapeutic role of Mdivi in ameliorating heart failure.

## Introduction

Heart failure is a multi factorial syndrome and is the leading cause of mortality worldwide [1]. Pressure overload created hemodynamic stress results in initial compensated cardiac hypertrophy and later leads to decompensated heart failure on sustained overload. Vast research has been going on to study the pathogenesis of heart failure including matrix metalloproteinases (MMPs), their tissue inhibitors (TIMPs) induced cardiac remodeling and role of anti angiogenic factors [2]. More recently several studies reported the evidence of cardiac autophagy in the pathogenesis of load induced heart failure [3], [4], [5], [6]. Autophagy is a lysosomal mediated process scavenging the old nonfunctional and damaged cellular structures like mitochondria, peroxisomes and maintains the homeostasis of cellular environment [7], [8], [9]. Though basal level autophagy is required, stress induced abnormal autophagy can be detrimental. A selective form of autophagy through which damaged mitochondria are removed by lysosomal degradation is termed as mitophagy [10], [11].

The dynamic process of myocyte contraction and relaxation is continuous energy demand process. Cardiac myocytes are highly packed with mitochondria and heart relies mostly on mitochondrial metabolism for its energy needs [12], [13]. Mitochondrial structural abnormalities along with the decreased levels of high energy phosphates are reported in cardiac myocytes of advanced heart failure stage [12], [14], [15]. Mitochondria are the main source of reactive oxygen species (ROS) that are involved in the pathogenesis of heart failure [16]. ROS, produced by mitochondria during stress can be detrimental to their own lipids, proteins and DNA and lead to mitochondrial dysfunction [17]. Increased ROS lead to loss of mitochondrial membrane potential (ΔΨm), which later signals for mitochondrial fragmentation (fission) and mitophagy [10], [11], [18].

Mitochondrial dynamics involve fission and fusion mechanisms regulated by enzymes that hydrolyze guanidine triphosphates (GTPases) [19]. Dynamin related protein (Drp1) and fission protein (hfis1) are mainly involved in mitochondrial fission mechanism [20], [21], whereas mitofusins 1 and 2 help in fusion mechanism [22], [23]. Activation of Drp1 is reported to stimulate the fission process during mitophagy [24], [25]. Mdivi-1 (Mitochondrial division inhibitor-1), a newly found potential inhibitor of Drp1 was reported to block the mitochondrial fission and confers cardio protection in experimental cardiac ischemia reperfusion studies in mice [26].

In the current study, we have created ascending aortic banding in mice to create pressure overload stress on the heart and later treated with potential mitochondrial division inhibitor (Drp-1 inhibitor), Mdivi-1 to study its effects on ventricular remodeling in heart failure. To our knowledge, we are the first to report the therapeutic role of Mdivi-1 during pressure overload in ameliorating the heart failure.

## Materials and Methods

### Animals

Wild type mice (WT, C57BL6/J) aged 8 weeks were procured from Jackson Laboratories (Bar Harbor, Me.; USA) and housed in the animal care facility at University of Louisville with access to standard chow and water. Ascending aortic banding was created in mice of 12 weeks age with an approximate weight of 23–25 grams. Each group is further divided into sham, AB 8 weeks treated with vehicle control and Mdivi. After the study period animals were euthanized in accordance with National Institute of Health Guidelines for animal research and were reviewed and approved by the Institute Animal Care and use Committee of University of Louisville (IACUC # 07134).

### Pressure overload animal model

Ascending aortic banding was done as described previously [2], [27].Briefly, under sodium pentobarbital anesthesia, animals were intubated and ventilated with Harvard mini ventilator. Body temperature was maintained with a heating pad. Under sterile surgical environment thorax was opened by left parasternal thoracotomy and ascending aorta was dissected and separated from the adjacent structures. Ascending aorta was ligated with 6–0 silk by placing the 26 g needle on the aorta for optimum constriction. Needle was quickly removed to keep the constricted aorta patent. Wound was closed in layers using 6–0 vicryl for the subcutaneous tissues and 5–0 silk to the skin. The mortality rate was less than 20% with the surgery. All animals were given post-operative analgesia with intraperitoneal injection of Ketofen, 5 mg/Kg body weight. Sham group of animals underwent similar procedure except the aortic constriction. By creating pressure overload using ascending aortic banding model, heart failure develops by 8 weeks. Our previous experiments showed the pathophysiological and histological changes associated with heart failure are achieved by 8 weeks [2], [27]. Researchers showed the therapeutic effects of Mdivi in ischemia reperfusion studies suggesting that it prevents heart failure [26]. Here we show that Mdivi can alleviate left ventricular dysfunction during heart failure condition.

### Mdivi treatment

Mitochondrial division inhibitor, Mdivi-1 was purchased from Enzo life sciences (Plymouth meeting, PA). Mdivi-1 was given as intraperitoneal injection in the dose of 50 mg per kg body weight for 7 days. Since Mdivi was dissolved in di-methyl sulfoxide (DMSO), we gave similar dilution of DMSO in the normal saline as vehicle control. At the end of the treatment, animals were euthanized and organs were harvested and stored at −80°c.

### Antibodies & Reagents

The following primary antibodies were used for immunohistochemical data: rabbit polyclonal anti-angiostatin, mouse monoclonal anti-endostatin, rabbit polyclonal MMP-2, rabbit polyclonal MMP-9, rabbit polyclonal antibody against TIMP-3, LC3, mouse monoclonal mitochondrial marker- MTCO-2 and mouse monoclonal P62 antibodies. These antibodies were purchased from Abcam (Cambridge, MA). Anti-mouse VEGF antibody was purchased from R&D systems (Minneapolis, MN). Anti-mouse CD 31 or PECAM (Platelet endothelial cell adhesion molecule) was purchased from BD Pharmingen (Sandiego, CA). Cleaved caspace-3 antibody (Cell Signaling #9661) from rabbit source was used. Following fluorescent secondary antibodies for Immunohistochemistry (IHC) were ordered from Invitrogen (Carlsbad, CA): Texas Red raised in mouse, Alexa Fluor 488 ,594 raised in rabbit and Alexa fluor 647 raised in rat.

### Echocardiography

Left ventricular functional status was assessed by transthoracic echocardiography as described elsewhere [27]. Briefly, echo was performed on mice to achieve two dimensional left ventricle images from an apical view using a SONOS 5500 or 2500; Hewlett-Packard, Inc. and a 12.5 MHz transducer. Tribromo ethanol (TBE) anesthesia (intra peritoneal dose of 240 mg/kg body weight), was used to minimize the cardio depressing actions produced by other anesthetics [28]. Mice were depilated with hair removal cream (Nair) and placed on a heating pad to maintain body temperature. The functional status of the heart was assessed by LVIDd, LVIDs, LVPWD and %FS. %FS is the most common method to evaluate left ventricular function in murine echocardiography [29].

### Cryosectioning

After euthanizing the mice, heart tissue was harvested and washed thoroughly in phosphate buffered saline (PBS) and preserved in a Peel-A-Way disposable plastic tissue embedding moulds (Polysciences inc., Warrington, PA.,USA) having tissue freezing media (Triangle Biomedical Sciences, Durham, N.C., USA) at 70° C until further use. 5 µm thickness tissue sections were made using Cryocut (Leica CM 1850) and placed on Super frost plus microscope slides, air-dried and processed for staining.

### Immunohistochemistry

5 µm thick frozen sections of the heart were used to perform immunohistochemistry (IHC) following standard IHC protocol (Abcam) as described previously [2]. Following overnight primary antibody application, secondary antibodies were applied as described in the protocol and stained slides were mounted and visualized with fluorescence by a laser scanning confocal microscope (Olympus FluoView1000) with an appropriate filter.

### Tunel assay

Tunel staining was conducted on frozen heart sections using a commercially available kit (Dead End Fluorometric TUNEL System; Promega). Staining was done according to manufacturer's instructions keeping positive and negative controls.

### Masson's Trichrome Staining

Fibrosis or collagen deposition in tissue sections was assessed by Mason's trichrome (Richard-Allan Scientific, Kalamazoo, MI, USA) staining as per the manufacturer's instructions. Collagen appeared as blue color.

### Image Proplus software

Images from Immunohistochemistry and Masson's trichrome staining were analyzed with Image Proplus software. Image analysis was done using image-pro software in confocal images taking different optical fields at random into consideration while the densitometry analysis was done using Bio-Rad software.

### RNA extraction and quality assessment

Total RNA from mouse heart was isolated using Trizol reagent (Gibco BRL) following manufacturer's instructions. RNA quality was assessed by Nano Drop ND-1000 and only highly pure quality RNA (260/280–2.00 and 260/230–2.0) was used for RT-PCR studies.

### RT-PCR

cDNA was prepared using promega kit. The reverse transcription program was 25°C for 10 min, 42°C for 50 min, and then 70°C for 15 min. RT-PCR was performed for mRNA expression of MMP-2,-9,TIMP-3, VEGF, angiostatin, endostatin in study groups using ImProm-II™ Reverse Transcription system kit (Promega Corporation, Madison, WI, USA, cat # A3800). For gene amplification the RT-PCR program was 95°C–7.00 min, [95°C–0.50 min, 55°C–1.00 min, 72°C–1.00 min]×34, 72°C–5.00 min, 4°C-∞. For VEGF, the RT-PCR program was 95° C-7.00 min, [95°C–0.50 min, 63°C–1.00 min,95°C- 0.50 min,63°C-1.00 min, 72°C–1.00 min]×34, 72°C–5.00 min, 4°C-∞. The primers for RT-PCR are obtained from Invitrogen (carlsbad,CA) and described in Table 1. The RT-PCR product was electrophoresed on 1% agarose gel in TAE with 0.008% ethidium bromide.

**Table 1:** mRNA primers.

mRNA	Orientation	Primer Sequence
Endostatin	Forward	5-GTGGGTAACCTTTCTCCTCC-3
	Reverse	5-GGTTGACGATGGGCACAGAC-3
Angiostatin	Forward	5-GAGGTCAAGGTTGCCATGTT-3
	Reverse	5-GTTGCTTGCTTTCCCTCTTG-3
VEGF	Forward	5-GGACCCTGGCTTTACTGC-3
	Reverse	5-CGGGCTTGGCGATTTAG-3
MMP-2	Forward	5-CACACCAGGTGAAGGATGTG-3
	Reverse	5-GCCCTCCTAAGCCAGTCTCT-3
MMP-9	Forward	5-TAGTGAGAGACTCTACACAG-3
	Reverse	5-CCACTTCTTGTCAGTGTCGA-3
TIMP-3	Forward	5-GCAGATGAAGATGTACCGAGG-3
	Reverse	5-TCAGGGATCTGTGGCGTTGCT-3
GAPDH	Forward	5-ATGGGAAGCTGGTCATCAAC-3
	Reverse	5-TGTGAGGGAGATGCTCAGTG-3

### Quantitative real time PCR

cDNA obtained as mentioned above method was amplified by real time PCR method using Sybr green dye. Gene amplification program used was “95°C-10.00 min, 95°C-0.30 min, 58°C-1.00 min×40, 72°C-0.30 min, 95°C- 1.00 min, 55°C-0.30 min, 95°C-0.30 min.

### Statistical analysis

All data are expressed as mean ± SE. One-way analysis of variance (ANOVA) was performed to test for treatment effects, and differences between groups were determined using Tukey's post-hoc test. A p value <0.05 was considered to be significant.

## Results

### Left ventricular dysfunction was ameliorated by treatment with mitochondrial fission inhibitor

**Figure 1:**
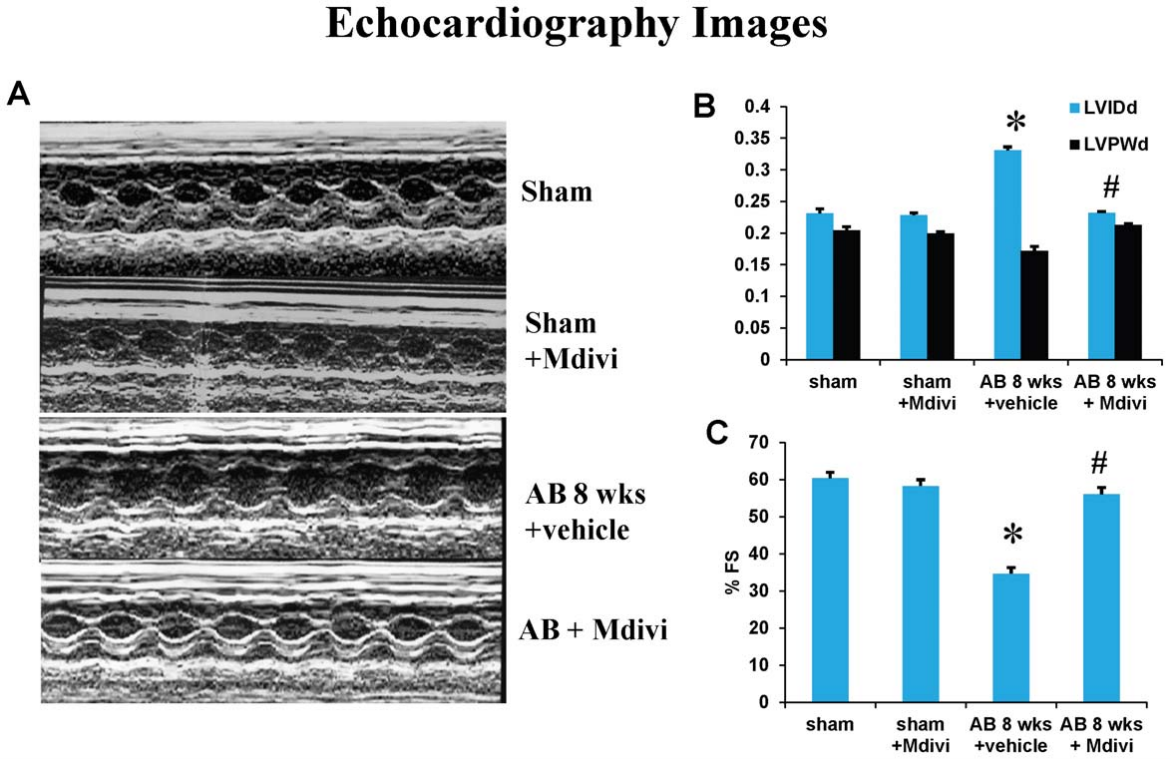
Mdivi ameliorates left ventricular dysfunction. **A**) Changes in left ventricular (LV) function following aortic banding (AB) and the effects of Mdivi treatment, a Drp1 inhibitor. Representative M-mode echocardiography images from sham, sham with Mdivi treatment, AB 8 weeks with vehicle and Mdivi treatment groups. The bar graphs represent LVIDd (left ventricular internal dimension in diastole), LVPWd (left ventricular posterior wall dimension in diastole (**B**), and %FS (percentage fractional shortening) (**C**). *p<0.05 compared to sham and ^#^p<0.05 compared to vehicle treated group. Data represents mean ±SE from n = 6 per group.

cardiac functional status was evaluated by echocardiography to determine the effect of Mdivi on left ventricular dysfunction. Echocardiography results showed a decrease in ventricular chamber diameter and also increase in percentage fractional shortening (%FS) in Mdivi treated AB 8 weeks group compared to AB 8 weeks vehicle control group (Figure 1). These findings suggest that, inhibiting the mitochondrial fragmentation using Mdivi helps in ameliorating ventricular dysfunction during pressure overload induced heart failure. To verify any side-effects of M-divi treatment, sham animals were treated with Mdivi and left ventricular function by echocardiography was assessed. Based on previous research studies [26] and from our findings, Mdivi didn't exhibit any side effects.

### Drp-1 inhibitor, Mdivi promotes angiogenesis

**Figure 2:**
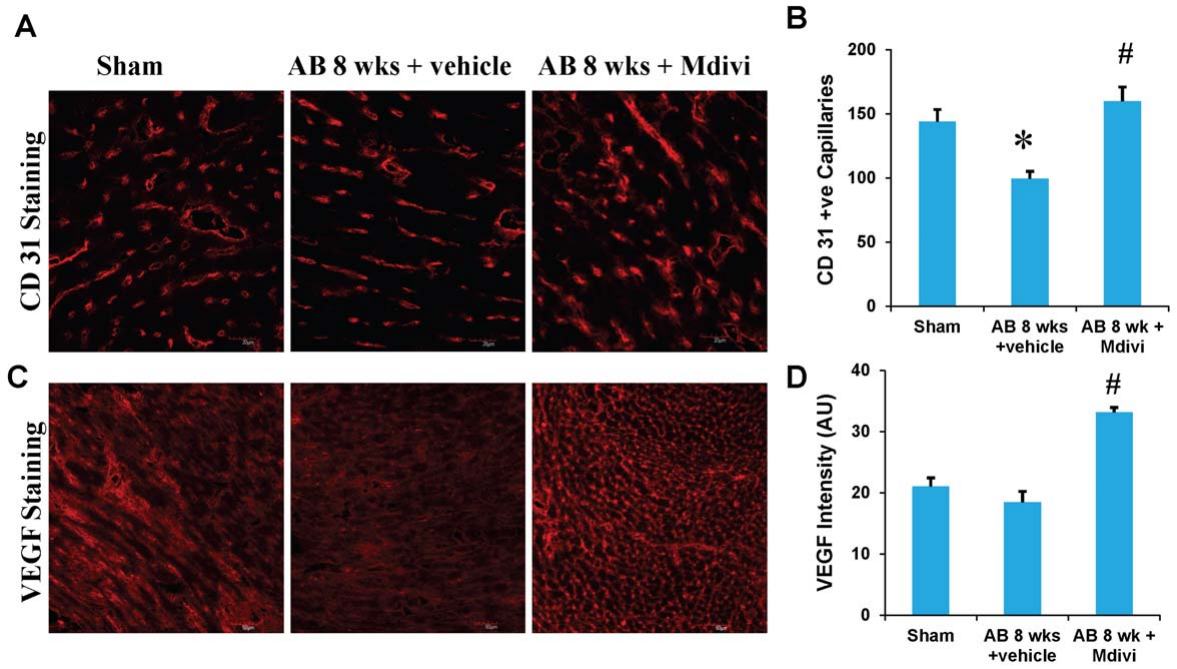
Mdivi promotes angiogenesis. **A**) CD-31-immunohistochemical (IHC) staining of heart and secondarily stained with alexaflour 647 in sham, 8 weeks post-AB (AB 8 wks) treated with vehicle control and Mdivi. The expression of CD31 is seen as red fluorescence intensity (scale bar- 20 µm). **C**) VEGF-IHC staining of heart sections, secondarily stained with alexaflour 594 in sham, 8 weeks post-AB (AB 8 wks) treated with vehicle control and Mdivi. The expression of VEGF is seen as red fluorescence intensity (scale bar- 50 µm). **B, D**) Data represents mean ±SE from n = 6 per group; *p<0.05 compared to sham and ^#^p<0.05 compared to vehicle treated group.

Angiogenesis was assessed by the expression of CD 31 or PECAM (Platelet endothelial cell adhesion molecule), an angiogenesis marker and by the expression of vascular endothelial growth factor (VEGF). Immunohistochemical data showed increase in the CD31 stained capillaries in Mdivi treated group compared to corresponding vehicle treated group. Increase in VEGF expression also confirms this finding and suggests that minimizing the abnormal mitophagy by Mdivi helps in angiogenesis during heart failure condition (Figure 2).

### Mdivi reduces the expression of anti-angiogenic factors and ameliorates ventricular remodeling during heart failure

**Figure 3:**
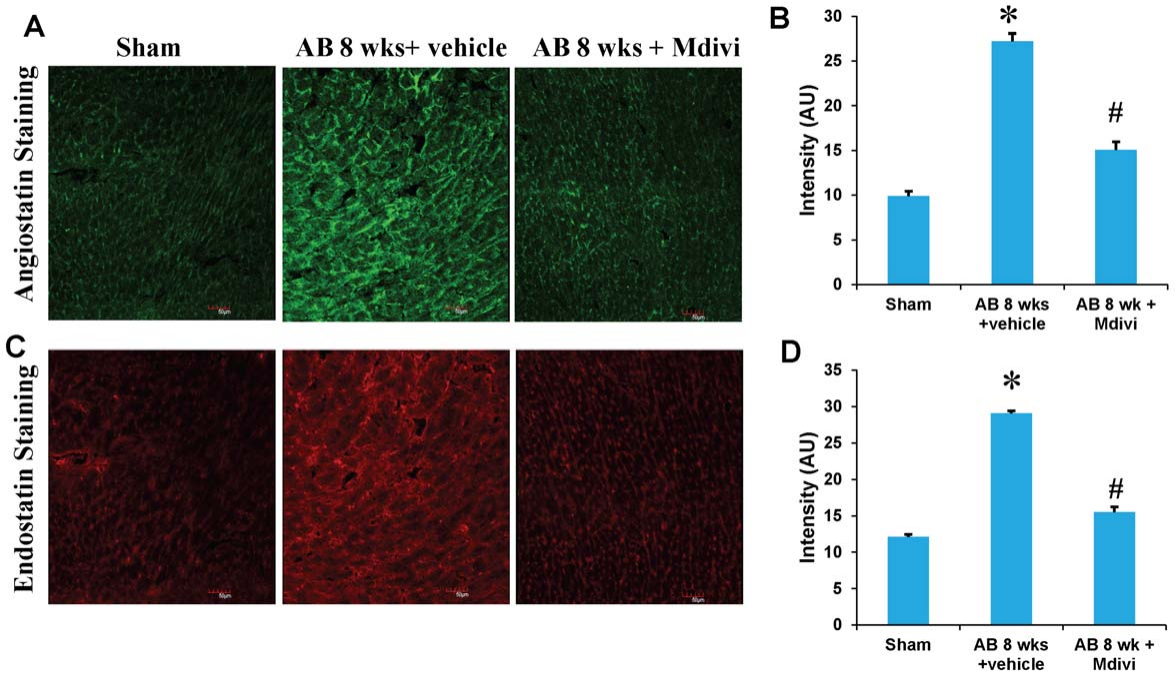
Mdivi inhibits anti-angiogenic factors. **A**) IHC staining of heart sections with angiostatin, secondarily stained with alexa fluor 488 fluorescent antibody in sham, 8 weeks post-AB (AB 8 wks) treated with vehicle control and Mdivi. The expression of angiostatin is seen as green fluorescence intensity (scale bar- 50 µm). **C**) IHC staining of heart sections with endostatin, secondarily stained with texas red fluorescent antibody in sham, 8 weeks post-AB (AB 8 wks) treated with vehicle control and Mdivi. The expression of endostatin is seen as red fluorescence intensity (scale bar- 50 µm). **B, D**) Data represents mean ±SE from n = 6 per group; *p<0.05 was considered significant compared to sham and ^#^p<0.05 compared to vehicle treated group.

**Figure 4:**
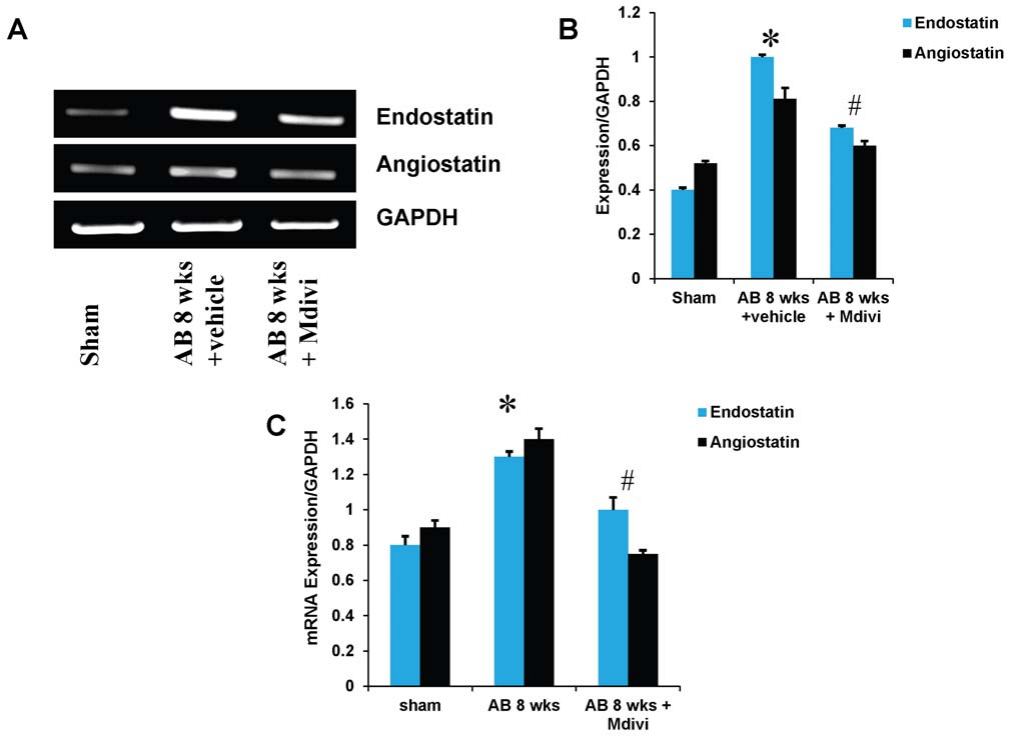
mRNA expression of endostatin and angiostatin primers. **A**) RT-PCR: mRNAs are amplified using respective primers and the bands were quantified using densitometry. **B, C**) Bar graphs represent respective mRNA expression over GAPDH expression by RT-PCR and real time PCR assay. Data represents mean ±SE from n = 6 per group; *p<0.05 was considered significant compared to sham and ^#^p<0.05 compared to vehicle treated group.

To determine the role of Mdivi on anti-angiogenesis, expression of anti-angiogenic factors like angiostatin and endostatin were assessed by immunohistochemistry, RT-PCR and real time PCR. There was increased expression of angiostatin and endostatin in AB 8 weeks vehicle treated group compared to Mdivi treated group (Figure 3 & 4). These results suggest that inhibiting the mitochondrial fission by Mdivi can decrease the expression of anti-angiogenic factors.

### Mdivi regulates extra cellular matrix remodeling by altering MMP/TIMP expressions during heart failure

**Figure 5:**
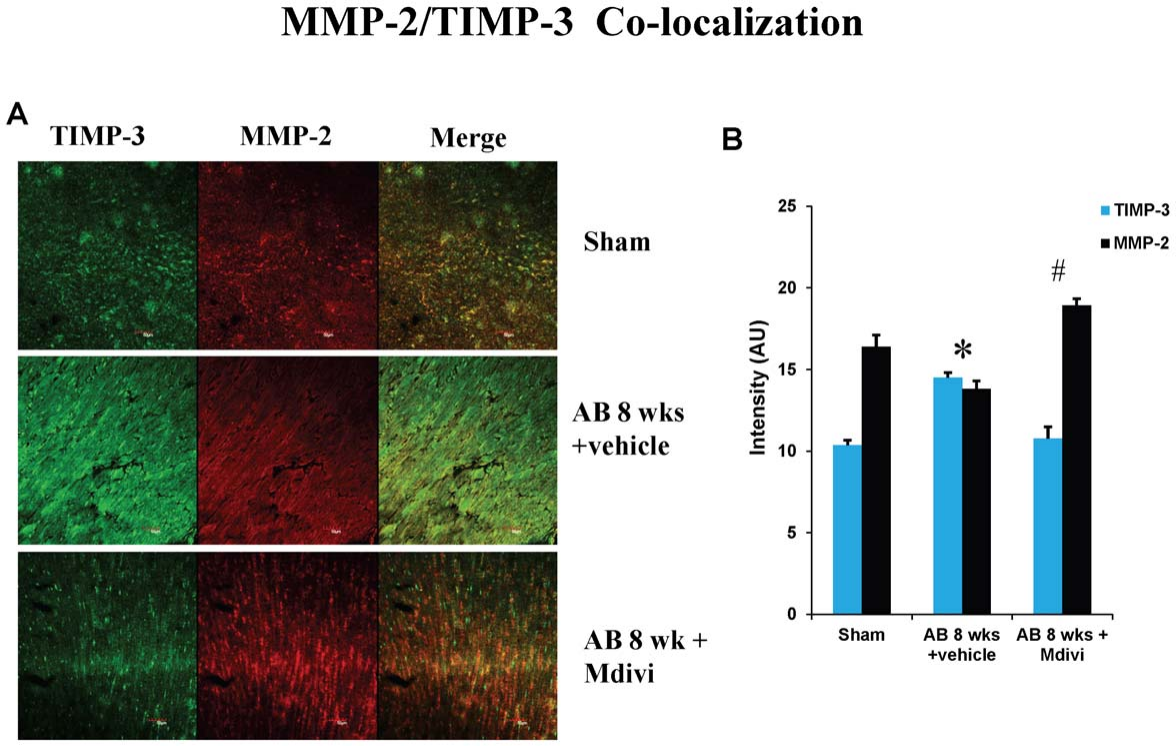
Co-localization staining of heart sections with MMP-2 and TIMP-3, secondarily stained with texas red and alexaflour 488 respectively, fluorescent antibodies in sham, in sham, 8 weeks post-AB (AB 8 wks) treated with vehicle control and Mdivi. **A**) The expression of MMP-2 is seen as red fluorescence intensity and TIMP-3 as green fluorescence (scale bar- 50 µm). **B**) Images were analyzed using Image proplus software and the data represented in bar diagram. Data represents mean ±SE from n = 6 per group; *p<0.05 was considered significant compared to sham and ^#^p<0.05 compared to vehicle treated group.

**Figure 6:**
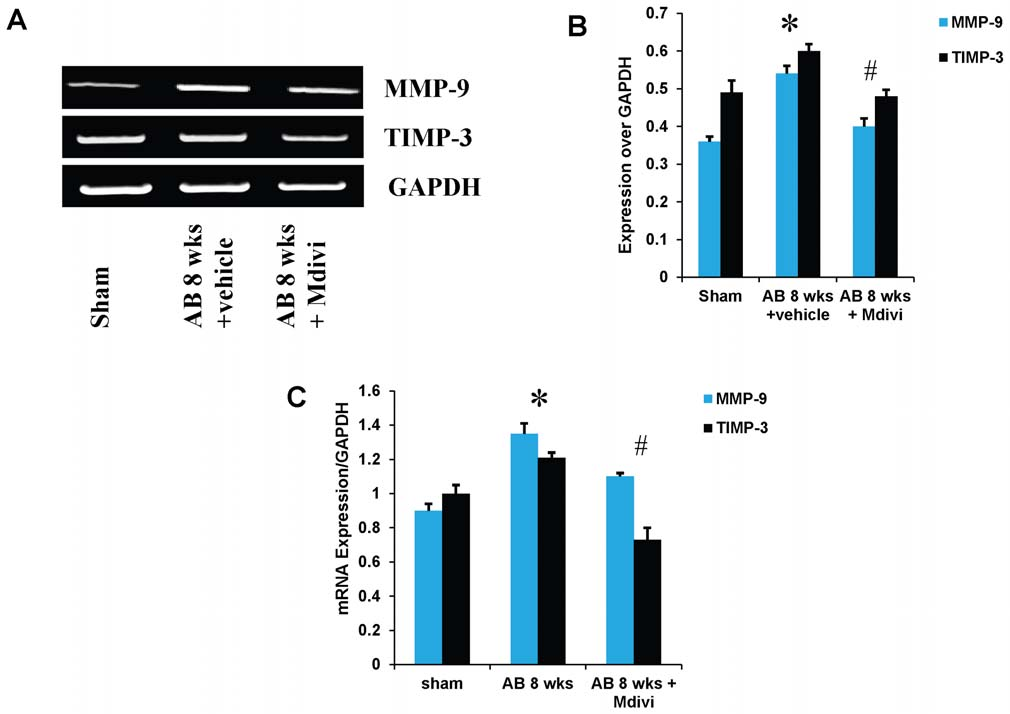
mRNA expression of MMP-9 and TIMP-3. A) mRNAs are amplified using respective primers and the bands were quantified using densitometry. **B, C**) Bar graphs represent respective mRNA expression over GAPDH expression by RT-PCR and real time PCR assay. Data represents mean ±SE from n = 6 per group; *p<0.05 was considered significant compared to sham and ^#^p<0.05 compared to vehicle treated group.

**Figure 7:**
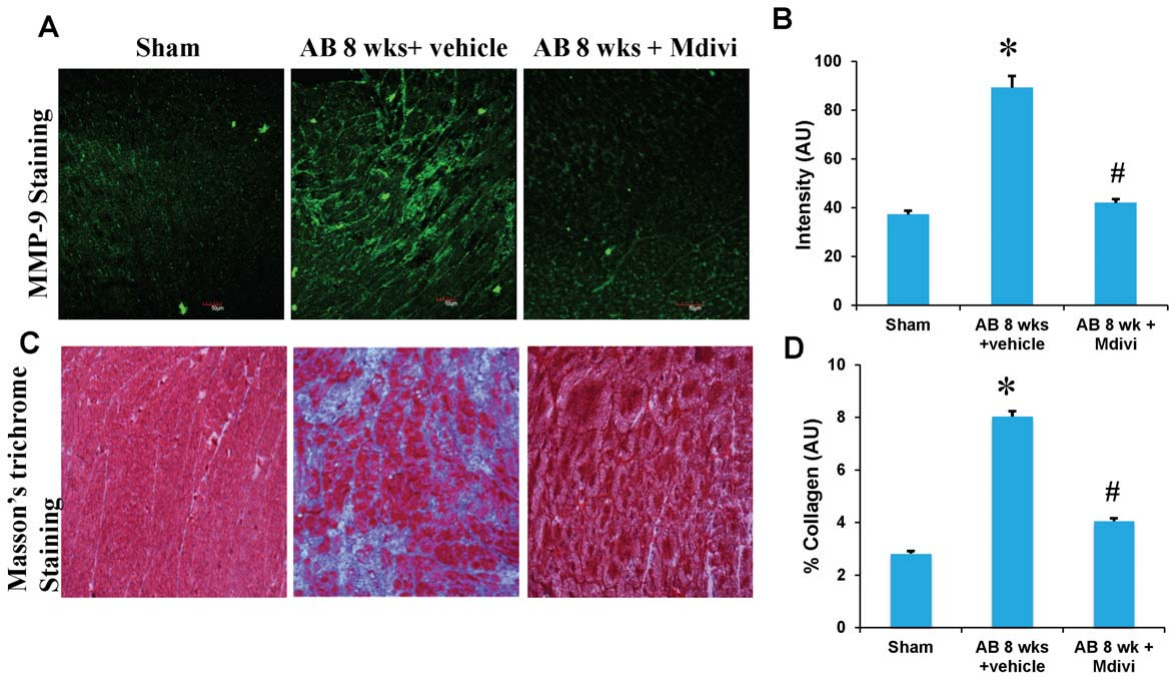
IHC staining of heart sections with MMP-9 antibody, secondarily stained with alexaflour 488 fluorescent antibody in sham, 8 weeks post-AB (AB 8 wks) treated with vehicle control and Mdivi. **A**) The expression of MMP-9 is seen as green fluorescence intensity (scale bar- 50 µm). **C**) Masson's trichrome blue staining of collagen depicting interstitial fibrosis. Quantification of collagen (blue staining) was done with the help of Image proplus software and represented in bar diagram. **B, D**) Data represents mean ±SE from n = 6 per group; *p<0.05 was considered significant compared to sham and ^#^p<0.05 compared to vehicle treated group.

To assess the role of Mdivi on MMP/TIMP axis, expression of MMP-2,-9 and TIMP-3 were determined by immunohistochemistry, RT-PCR and real time PCR. There was increased expression of MMP-9 and TIMP-3 in AB 8 weeks vehicle treated group compared to sham. Treatment with Mdivi decreased the expression of MMP-9 and TIMP-3. In contrast, MMP-2 expression in IHC data showed increase in Mdivi treated group compared to corresponding vehicle treated group (Figure 5, [6] & [7]). These findings suggest that Mdivi regulates abnormal ventricular remodeling by altering the MMP/TIMP axis during pressure overload induced heart failure. Previously, we and others have shown the association of MMP-9 and TIMP-3 with collagen deposition and fibrosis [2], [27]. Thus we investigated the role of Mdivi in collagen deposition or fibrosis through well-established Masson's trichrome staining. There was significant increase in collagen deposition in vehicle treated AB 8 weeks group compared to Mdivi treated and sham groups (Figure 7). These findings suggest that treatment with Mdivi reduces fibrosis or collagen deposition.

### Mdivi prevents abnormal apoptosis

**Figure 8:**
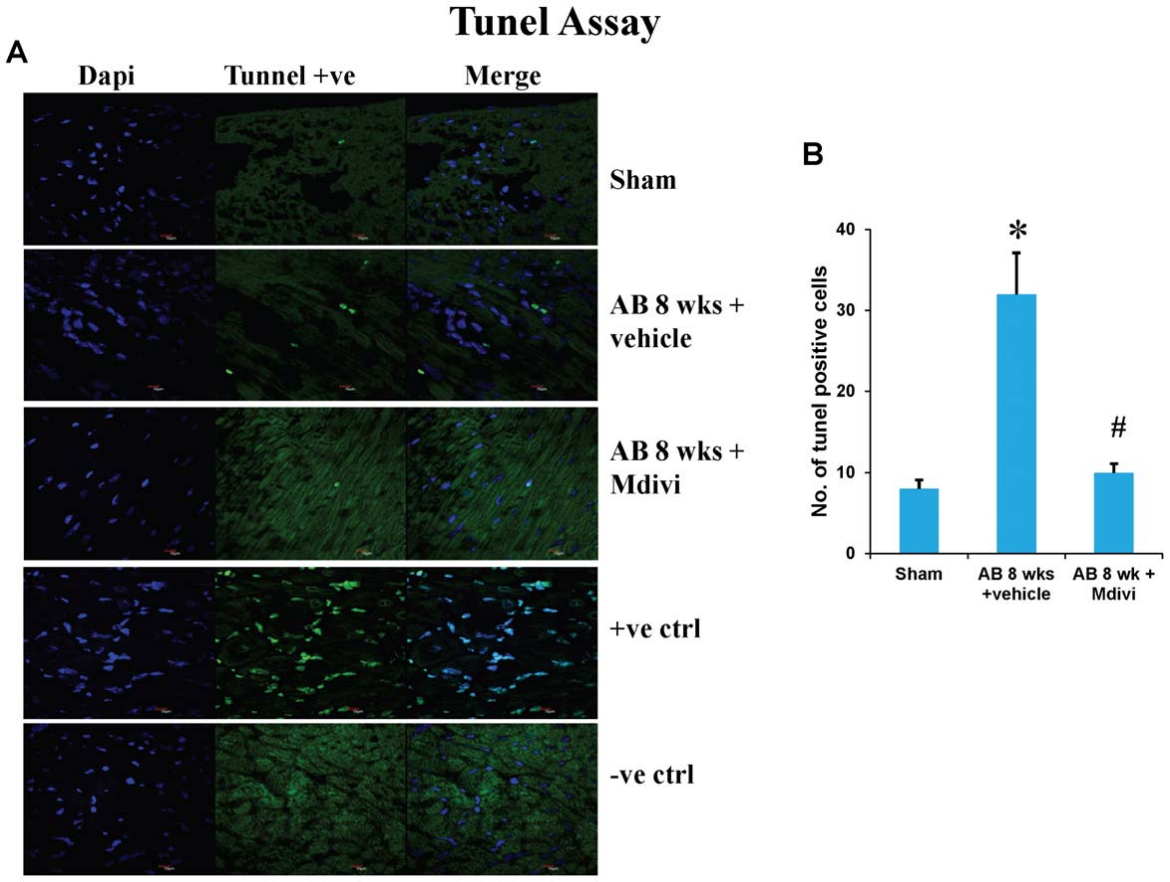
Tunnel assay of mouse cardiac sections staining for apoptosis in sham, 8 weeks post-AB (AB 8 wks) treated with vehicle control and Mdivi. **A**) Apoptotic cells are seen as green fluorescent dots (scale bar- 10 µm). Positive and negative controls are also represented in this image. **B**) Data represents mean ±SE from n = 6 per group; *p<0.05 was considered significant compared to sham and ^#^p<0.05 compared to vehicle treated group.

**Figure 9:**
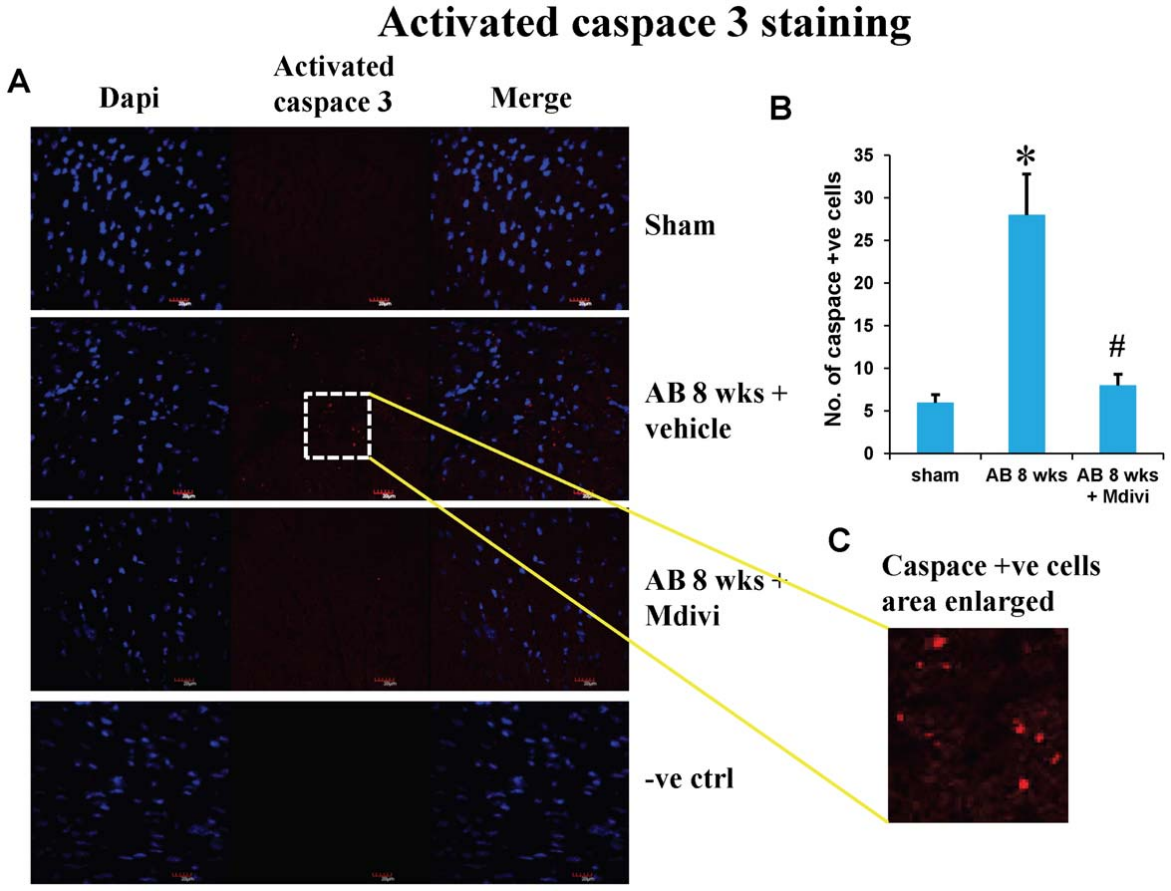
Activated caspace-3 staining of mouse cardiac sections showing apoptosis in sham, 8 weeks post-AB (AB 8 wks) treated with vehicle control and Mdivi. **A**) Apoptotic cells are seen as red fluorescent dots (scale bar- 20 µm). **C**) Activated caspace-3 +ve cells represented in an enlarged area. **B**) Data represents mean ±SE from n = 6 per group; *p<0.05 was considered significant compared to sham and ^#^p<0.05 compared to vehicle treated group.

Apoptosis or cell death during heart failure was assessed by Tunel assay and activated caspace-3 staining. There was significant increase in apoptosis level in AB 8 weeks vehicle treated group. Treatment with Mdivi inhibits the increase in apoptosis and normalizes to that of sham group (Figure 8 & 9). These findings suggest the beneficial role in preventing apoptosis.

### Treatment with Mdivi minimizes the expression of autophagy/mitophagy markers and increases the expression of mitochondrial marker MTCO-2

**Figure 10:**
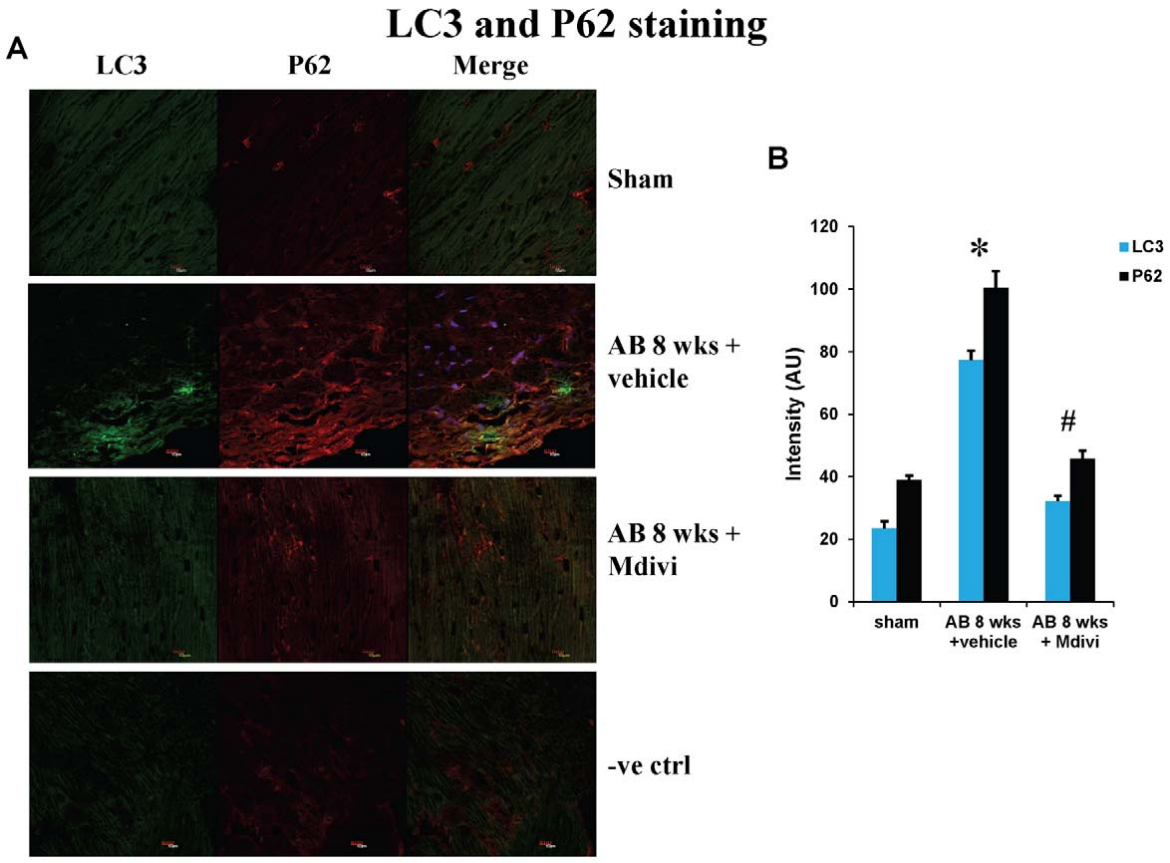
IHC staining of heart sections with mitophagy markers LC3 and P62 in sham, 8 weeks post-AB (AB 8 wks) treated with vehicle control and Mdivi. **A**) Expression of LC3 is seen as green fluorescence intensity and P62 as red fluorescence intensity (scale bar- 10 µm). **B**) Data represents mean ±SE from n = 6 per group; *p<0.05 was considered significant compared to sham and ^#^p<0.05 compared to vehicle treated group.

**Figure 11:**
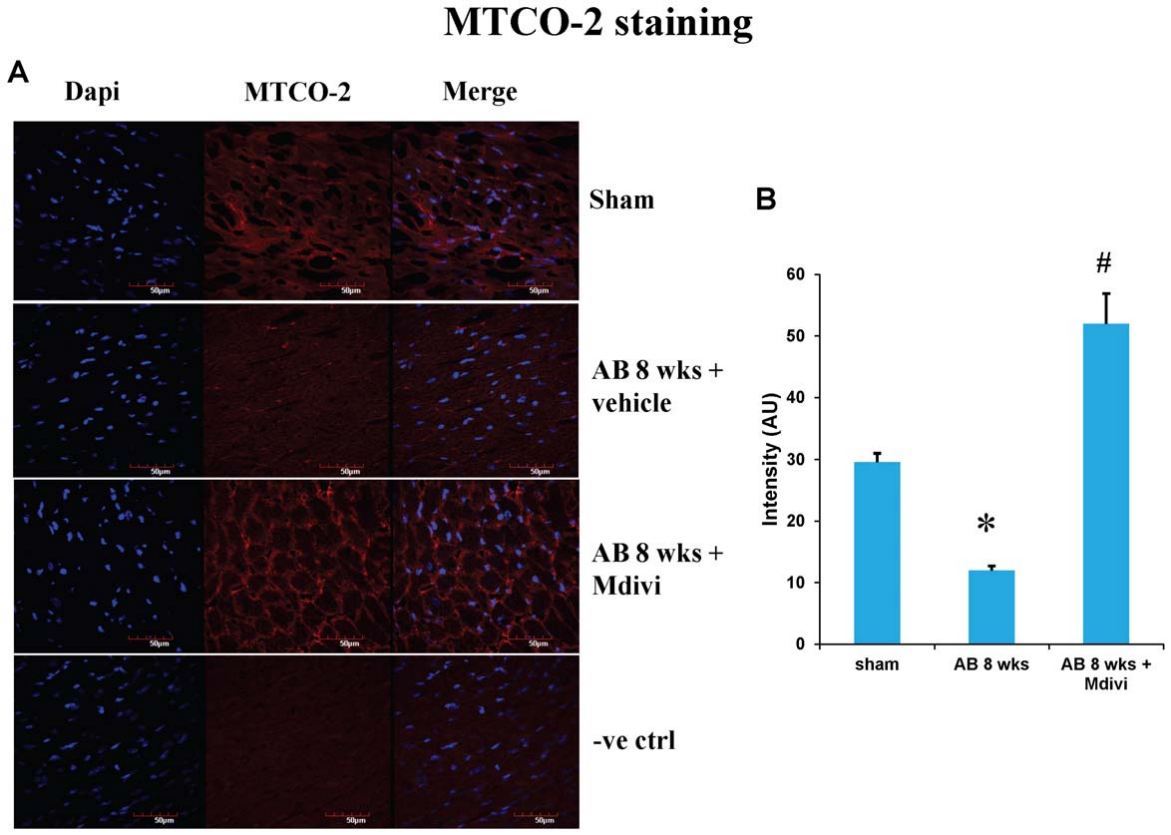
IHC staining of mouse heart sections with mitochondrial marker antibody MTCO-2 in sham, 8 weeks post-AB (AB 8 wks) treated with vehicle control and Mdivi. **A**) Expression of MTCO-2 is seen as red fluorescence intensity (scale bar-50 µm). **B**) Data represents mean ±SE from n = 6 per group; *p<0.05 was considered significant compared to sham and ^#^p<0.05 compared to vehicle treated group.

Autophagy/Mitophagy in heart failure was assessed by expression of mitophagy markers like LC3 and P62. There was increase in expression of LC3 and P62 in AB 8 weeks vehicle treated group compared to sham. Treatment with mitochondrial fission inhibitor, Mdivi normalized the increased expression of LC3 and P62 to that of sham group (Figure 10). Expression of mitochondrial marker MTCO-2 is increased significantly in Mdivi treated groups compared to untreated groups suggesting the increased number of mitochondria (Figure 11). These results suggest that Mdivi inhibits the abnormal mitophagy, restores the number of mitochondria and helps in ameliorating heart failure condition.

## Discussion

Previous studies reported the induction of autophagy during pressure overload induced heart failure. Mitochondria being the main source of cellular reactive oxygen species (ROS), are the main target for autophagy and thus mitophagy is increased during end stage of heart failure. Studies also reported that Drp-1 inhibitor, Mdivi inhibits mitochondrial fission during mitophagy and confers cardio protection during ischemia-reperfusion. The purpose of this study was to determine the therapeutic role of Mdivi during pressure overload induced heart failure.

We have previously reported the role of matrix metalloproteinases induced angiogenesis during pressure overload induced stress in initiating compensated cardiac hypertrophy and the role of anti angiogenic factors leading to decompensated heart failure on sustained overload [2], [27]. The rationale and hypothesis of this study were that Mdivi primarily inhibits mitochondrial fission and thereby inhibits the mitophagy/autophagy during pressure overload induced heart failure. During this process Mdivi regulates abnormal ventricular remodeling by altering MMP/TIMP axis, inhibits expression of anti angiogenic factors and also promotes angiogenesis, thus ameliorates heart failure. In our current study, we found that there is increased induction of cardiac autophagy/mitophagy and also there is increased apoptosis during heart failure stage. Finally, treatment with Drp-1 inhibitor, Mdivi-1 confers cardio protection by inhibiting the mitochondrial fragmentation that is increased during heart failure.

Heart disease is the number one cause of mortality worldwide. Recent studies report the role of autophagy or mitophagy in the pathogenesis of heart failure [3]. Though mitochondrial dynamics involve both fission and fusion mechanisms, mitochondrial fragmentation or fission signals the process of mitophagy [19], [30], [31], [32]. Mdivi is the known potent Drp-1 inhibitor and has been reported of its protective role in mice models of experimental ischemia reperfusion studies in both heart and kidneys [26], [33], [34]. The significant increase in ventricular chamber diameter and decrease in fractional shortening suggests the ventricular dysfunction during AB 8 weeks stage. Functional studies by echocardiography showed that treatment with Mdivi ameliorates the left ventricular dysfunction in AB 8 weeks group (Figure 1), which is further supported by other immunohistochemical and molecular data.

Studies report that imbalance in capillary-myocyte ratio is the prime cause of transition from compensated hypertrophy to heart failure [35], [36]. We and others showed that increasing the angiogenesis during pressure overload hemodynamic stress protects the heart from injury [27], [37]. Also, endostatin was shown to be an inducer of autophagy [38]. Here we report that treatment with Mdivi, not only induces angiogenesis by increasing expression of CD31 and VEGF but also decreases the expression of anti angiogenic factors (Figure 2, 3 & 4). MMPs and TIMPs play an important role in inducing ventricular remodeling during cardiac hemodynamic overload. Studies have shown the pro angiogenic role of MMP-2 and anti angiogenic role of MMP-9 and TIMP-3 during this pathogenesis [2], [39], [40], [41], [42]. Besides, TIMP-3 was also shown to cause apoptosis [43]. By inhibiting the mitophagy, Mdivi increases the expression of pro angiogenic MMP-2 and decreases the expression of anti angiogenic MMP-9 and TIMP-3 in IHC data (Figure 5 & 6). Besides, mRNA expression of MMP-9 and TIMP-3 was significantly decreased in Mdivi treated groups (Figure 6). This suggests that decrease in expression of MMP-9 and TIMP-3 by Mdivi prevents collagen deposition as evidenced by the Masson's trichrome staining of heart sections (Figure 5, 6 & 7). Collagen content (fibrotic area) was analyzed in relation to entire myocardium excluding the pericardium and tips of papillary regions.

Apoptosis is defined as programmed cell death, while autophagy is eating of particular cellular organelles. Selective removal of mitochondria by autophagy is defined as mitophagy [44].Changes in mitochondrial morphology affects the cell susceptibility to apoptotic cell death and thus may involve in disease process [45]. Here we show increase in apoptosis by Tunel assay and activated caspace-3 staining during pressure overload induced failure stage compared to that of sham. Increase in expression of autophagy/mitophagy markers LC3 and P62 was also noticed in decompensated heart failure stage. Treatment with mitochondrial division inhibitor (Mdivi), inhibits abnormal mitophagy, thus significantly decreased apoptosis and also normalized expression of LC3, P62 to that of sham (Figures 8, 9 & 10). Direct effects of Mdivi on cardiomyocyte mitochondria were shown previously in myocardial ischemia reperfusion study [26].

### Conclusions

Our findings suggest that Drp-1 inhibitor, Mdivi-1 inhibits mitochondrial fission and thereby plays a role in a) ameliorating left ventricular dysfunction during heart failure, b) increases expression of CD31 and VEGF, thus angiogenesis, c) decreases expression of anti angiogenic factors and also minimizes collagen deposition, d) inhibits abnormal autophagy/mitophagy and also apoptosis.

### Limitations

This study being the first time to study the therapeutic effect of Mdivi in pressure overload induced heart failure, we have to optimize the dose and duration of the drug. From the previous literature in kidney reperfusion studies, it was reported that a dose of 50 mg/kg body weight was effective therapeutically. Although we couldn't look at the direct effect of Mdivi due to technical limitation, we showed the effect through mitophagy markers. Also we didn't measure the blood levels of the drug as we injected the drug intra peritoneal route directly.
